# Adrenaline, Takotsubo, Anaphylaxis, and Kounis Syndrome (ATAK) Complex Unveiled: Integrating Takotsubo and Kounis Syndromes in the Context of Chemotherapy-Related Anaphylaxis

**DOI:** 10.7759/cureus.53145

**Published:** 2024-01-29

**Authors:** Piyush Puri, Meet Popatbhai Kachhadia, Princy Sardana, Ridhi Bhagat, Sammir S Dekowski, Emmanuel Fohle

**Affiliations:** 1 Internal Medicine, Adesh Institute of Medical Science and Research, Bathinda, IND; 2 Internal Medicine, Pandit Deendayal Upadhyay (PDU) Medical College, Civil Hospital Campus, Rajkot, IND; 3 Internal Medicine, Saraswathi Institute of Medical Sciences, Hapur, IND; 4 Internal Medicine, Teerthanker Mahaveer Medical College and Reseach Center, Moradabad, IND; 5 Internal Medicine, Newark Beth Israel Medical Center, Newark, USA; 6 Internal Medicine, University of North Dakota, Fargo, USA

**Keywords:** atak syndrome, coronary hypersensitivity disorder, allergic angina, allergy, kounis case study, st elevation mi

## Abstract

The convergence of takotsubo and Kounis syndromes, collectively referred to as the "ATAK complex" (short for adrenaline, takotsubo, anaphylaxis, and Kounis syndrome), poses a unique and challenging clinical scenario, especially in the context of chemotherapy-related anaphylaxis. We present a case report involving a 63-year-old woman undergoing chemotherapy for endometrial adenocarcinoma who experienced anaphylactic symptoms during treatment. Immediate administration of epinephrine was followed by the emergence of ST elevation, a reduced left ventricular ejection fraction, and wall motion abnormalities indicative of stress-induced cardiomyopathy.

Detailed investigations revealed normal coronary arteries, prompting further exploration into the intricacies of the ATAK complex. Notably, the administration of intravenous rather than intramuscular epinephrine was identified as a contributing factor. This case underscores the critical importance of recognizing and managing the ATAK complex promptly, emphasizing the need for refined diagnostic and treatment guidelines. The interplay between adrenaline, takotsubo, anaphylaxis, and Kounis syndrome necessitates a nuanced approach, urging healthcare professionals to exercise caution and adhere to recommended administration routes. Increased awareness of the ATAK complex is imperative for optimizing patient outcomes and guiding therapeutic interventions in similar clinical scenarios. Further research is warranted to elucidate the underlying mechanisms and refine strategies for the effective management of this intricate syndrome.

## Introduction

Kounis syndrome is a subtype of coronary artery disease triggered by the body's reaction to allergens. It arises from a confluence of medications and environmental elements [1]. It is characterized as the simultaneous occurrence of acute coronary syndrome (ACS) and heightened sensitivity reactions resulting from an allergic episode [2]. Despite its peculiarities, this topic has rarely been described in the literature and has no guidelines for its diagnosis or treatment [2]. Kounis syndrome is a complex clinical condition that affects multiple organs and systems, including the coronary, cerebral, and mesenteric arteries, and is associated with allergic-hypersensitivity-anaphylaxis in the skin, respiratory, and vascular systems. It can arise in diverse medical settings, spanning anesthesia, surgery, radiology, oncology, dentistry, or psychiatry, with substantial implications for both morbidity and mortality [3]. The current approach to diagnosing Kounis syndrome heavily depends on clinical symptoms and patient history, potentially resulting in the inadvertent oversight or underdiagnosis of numerous cases. The introduction of Kounis syndrome was made by Kounis and Zavras in the year 1991, initially called "allergic angina" or "allergic myocardial infarction." In recent times, its association with takotsubo syndrome has been acknowledged as a part of the 'ATAK complex', which was named after the combination of adrenaline, takotsubo, anaphylaxis, and Kounis syndrome [4].

## Case presentation

**Figure 1:**
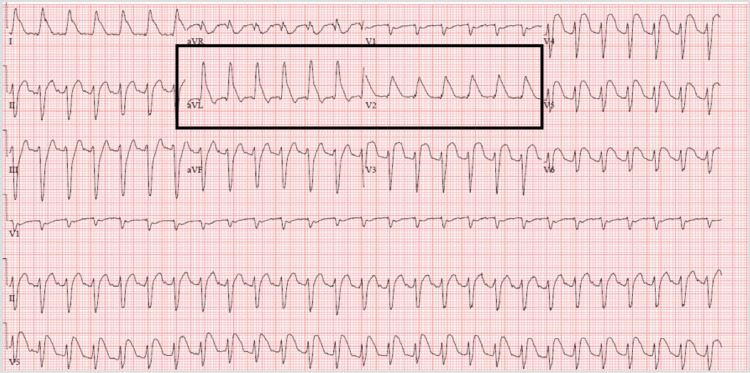
The EKG shows ST elevation with a shark fin pattern before treatment with epinephrine.

A 63-year-old woman who was recently diagnosed with endometrial adenocarcinoma was at the infusion center receiving the first dose of a chemotherapy regimen (carboplatin, paclitaxel, and pembrolizumab/placebo). After a few minutes of the infusion, the patient started complaining of chest pain, throat tightness, and shortness of breath. The patient became tachycardic and hypotensive. She was immediately treated with methylprednisolone, diphenhydramine, and epinephrine for a possible anaphylactic reaction. The electrocardiogram (ECG) showed ST elevation before treatment with epinephrine. (Figure 1).

**Figure 2:**
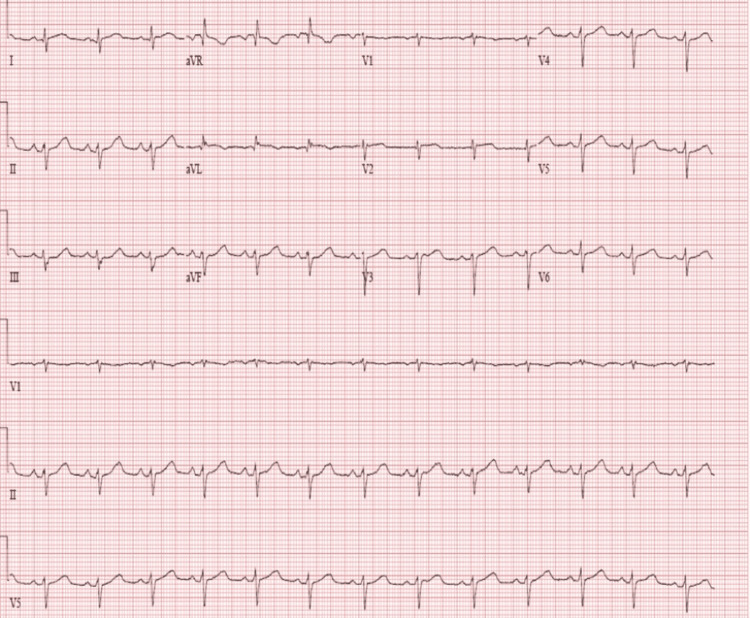
The EKG shows normalized ST elevation.

The ST-elevation myocardial infarction (STEMI) code was immediately activated. She was started on a saline bolus for severe hypotension. However, a few minutes later, after the treatment, the ECG showed a resolution of ST elevation (Figure 2).

Troponin I was assessed, which was 0.040 ng/mL initially. Every three hours, troponin I was trending as follows: 0.70 ng/mL > 0.040 ng/mL > 0.00 ng/mL. This denoted cardiac injury.

**Figure 3:**
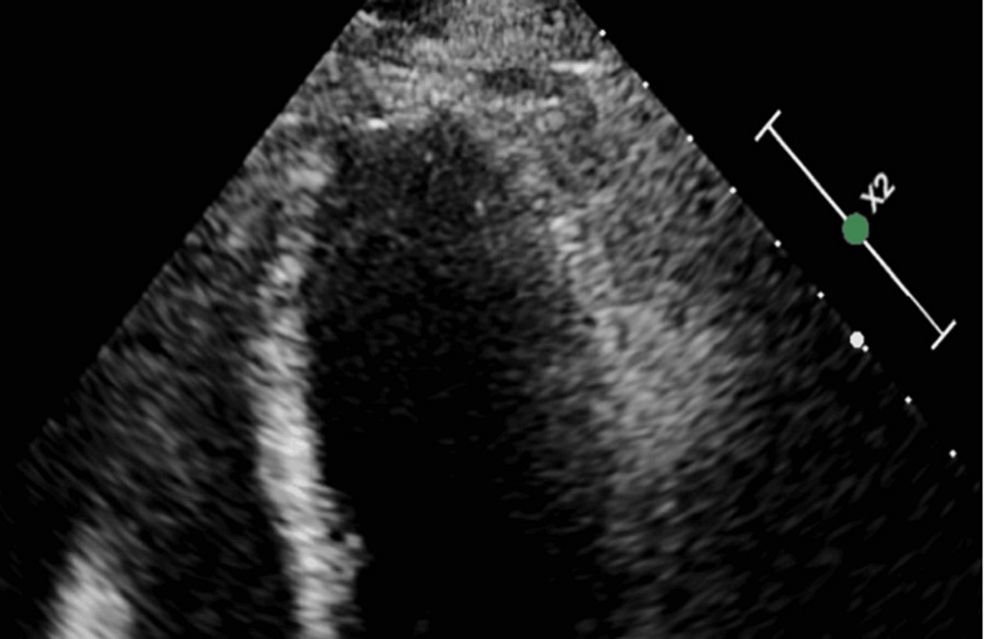
Echocardiogram with apical ballooning because of the anaphylaxis

Bedside echocardiogram (echo) showed a left ventricular ejection fraction of 35% with wall motion abnormalities suggestive of possible stress-induced cardiomyopathy (Figure 3).

**Figure 4:**
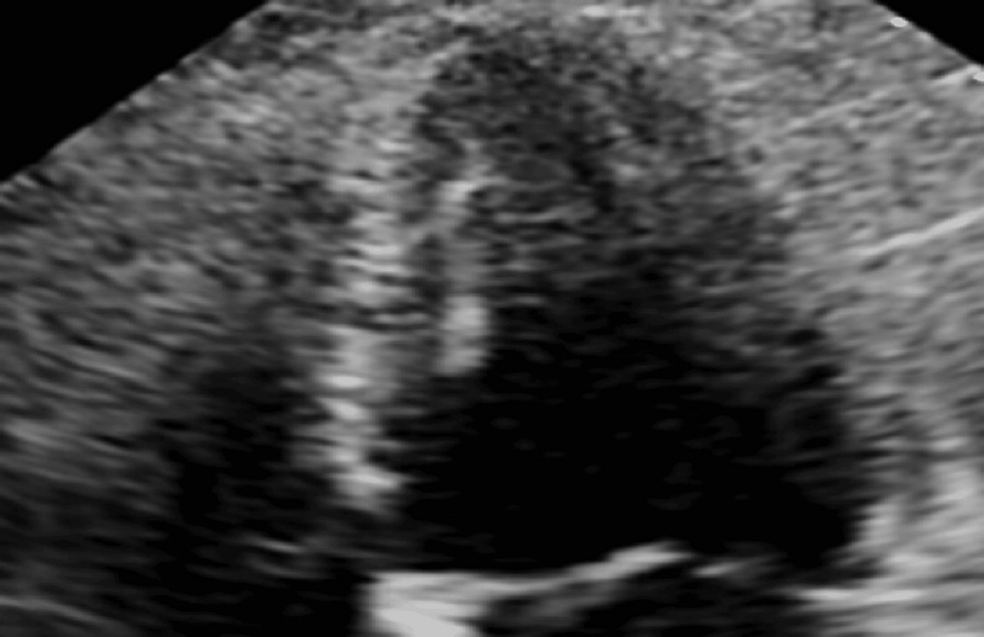
Reversal of apical ballooning

Because of the wall motion abnormalities, she underwent coronary angiography, which showed patent coronaries. Upon further review of the patient’s chart, it was noted that concentrated epinephrine (1:1000) was inadvertently administered intravenously. Repeat echos over the next few days showed normal systolic function (Figure 4).

It was postulated that the cause of ST elevation was secondary to Kounis syndrome.

This rare clinical case demonstrates how takotsubo and Kounis syndrome can come together in a single medical condition known as the ATAK complex. This entity requires specific management and has important implications for prognosis.

## Discussion

Kounis syndrome is a medical condition in which the heart experiences a lack of blood flow, known as myocardial ischemia or infarction, due to factors other than blood clots. Typically, this ailment is initiated by the release of inflammatory substances in response to an allergic or anaphylactic reaction. The primary mechanism involves the activation and disintegration of mast cells within the coronary arteries during such reactions. Consequently, chemical mediators such as histamine and tryptase are released, resulting in the constriction of the coronary arteries [5].

Takotsubo cardiomyopathy is a medical condition that takes its name from a Japanese fishing pot called "takotsubo" due to its resemblance to the left ventriculogram of affected patients. This condition is also known as transient left ventricular apical ballooning, stress-induced cardiomyopathy, takotsubo syndrome, apical ballooning syndrome, atypical apical ballooning, ampulla cardiomyopathy, broken heart syndrome, or transient left ventricular dysfunction syndrome. It primarily affects postmenopausal women after periods of emotional distress and presents with symptoms similar to acute myocardial infarction, including transient left ventricular dysfunction and mostly normal, but occasionally abnormal, coronary arteries. The exact cause, pathophysiology, and treatment of this condition are still unknown. According to the recently published Harmonizing Outcomes with Revascularization and Stents in Acute Myocardial Infarction (HORIZON-AMI) trial, the incidence of this condition is 2.1% in female patients and 0.5% in all patients with STEMI [6].

Various subtypes of Kounis syndrome have been identified in the literature. The majority of patients, accounting for 72.6%, belong to type I. These individuals experience coronary artery spasms triggered by inflammatory agents, despite having no clear stenosis. Type II, which accounts for 22.3% of Kounis syndrome patients, involves those with pre-existing coronary artery stenosis. Inflammatory agents cause coronary artery spasm in combination with atherosclerotic plaque rupture. Type III is additionally segmented into two specific subtypes. Type III-a, which accounts for 5.1% of Kounis syndrome patients, involves stent thrombosis, while type III-b involves stent restenosis after stent implantation [7, 8]. The primary diagnostic criteria for Kounis syndrome include a history of taking specific medications, allergic reactions, and cardiovascular symptoms. The diagnostic process involves conducting a variety of tests, including evaluations of serum tryptophan, histamine, cardiac enzymes, cardiac troponin, and trypsin, as well as utilizing techniques such as electrocardiograms, echocardiograms, and angiography [9].

More than seven decades have passed since Pfister et al. documented the initial case of myocardial infarction and urticaria following penicillin administration, yet recent case reports of Kounis syndrome continue to emerge. This suggests that its incidence is relatively low [8]. A significant epidemiological study in the United States reported 235,420 hospitalizations for anaphylaxis, hypersensitivity, or anaphylaxis. Among these patients, Kounis syndrome was identified in 2,616 individuals (11%) with ACS. In-hospital mortality was 7% overall (odds ratio (OR): 9.74, 95% CI: 8.08-11.76) [10]. There is no established treatment guideline for Kounis syndrome. Most physicians typically administer allergy and antiplatelet medications, manage vasospasm, perform coronary angiography, and endeavor to promptly open the affected vessel [11].

For anaphylactic shock, epinephrine is the first line of treatment, and antihistamines or glucocorticoids are used as a second option. The advantages and potential drawbacks of epinephrine use in clinical settings should be carefully weighed because it has the potential to cause malignant arrhythmia or coronary artery contraction [12]. The use of epinephrine is the preferred treatment for anaphylactic reactions due to its β-adrenergic properties, which result in bronchodilation, increased myocardial output, contractility, and the inhibition of further mediator release from mast cells and basophils. Additionally, its α-adrenergic properties lead to peripheral vasoconstriction. Epinephrine typically enhances coronary blood flow through its indirect vasodilatory effects, including prolonging diastole, elevating mean arterial pressure, and increasing the metabolic demands of the myocardium. The development of myocardial infarction with epinephrine use appears to involve an imbalance between its direct vasoconstrictive effects and its indirect vasodilatory effects on the coronary arteries, although the precise mechanism remains unclear [5].

Multiple studies have linked takotsubo cardiomyopathy to Kounis syndrome [13, 14]. It is important to further investigate the complex interplay between ATAK, as it presents a challenge in contemporary medicine [15]. Understanding the etiology and pathophysiology of this complex, as well as determining appropriate preventive and therapeutic measures, is crucial.

## Conclusions

This case report discusses a 63-year-old woman undergoing chemotherapy who experienced a unique medical condition termed the ATA complex, involving adrenaline, takotsubo, anaphylaxis, and Kounis syndrome. Following an episode of anaphylaxis and the inadvertent administration of intravenous epinephrine, the patient exhibited symptoms of takotsubo cardiomyopathy. The case underscores the importance of prompt recognition and management of this rare but potentially life-threatening condition. The discussion highlights the challenges in diagnosing and treating Kounis syndrome, emphasizing the need for increased awareness and caution in epinephrine administration. Further research is suggested to refine the diagnostic and treatment guidelines for this complex syndrome.

## References

[1] Ding P, Zhou Y, Long KL, Zhang L, Gao PY (2022). Case report: cefoperazone-sulbactam induced Kounis syndrome and cardiogenic shock. Front Cardiovasc Med.

[2] Omri M, Kraiem H, Mejri O, Naija M, Chebili N (2017). Management of Kounis syndrome: two case reports. J Med Case Rep.

[3] Kounis NG, Koniari I, Velissaris D, Tzanis G, Hahalis G (2019). Kounis syndrome—not a single-organ arterial disorder but a multisystem and multidisciplinary disease. Balkan Med J.

[4] Ballesteros RV, Polo JC, Olmos C, Vilacosta I (2023). Kounis and takotsubo, two syndromes bound by adrenaline: the “ATAK” complex. Case Rep Cardiol.

[5] Shrestha B, Kafle P, Thapa S, Dahal S, Gayam V, Dufresne A (2018). Intramuscular epinephrine-induced transient ST-elevation myocardial infarction. J Investig Med High Impact Case Rep.

[6] Prasad A, Dangas G, Srinivasan M, Yu J, Gersh BJ, Mehran R, Stone GW (2014). Incidence and angiographic characteristics of patients with apical ballooning syndrome (takotsubo/stress cardiomyopathy) in the HORIZONS-AMI trial: an analysis from a multicenter, international study of ST-elevation myocardial infarction. Catheter Cardiovasc Interv.

[7] Tsigkas G, Chouchoulis K, Theodoropoulos K, Kounis NG, Alexopoulos D (2011). Allergic reaction reveals a non-lethal late stent thrombosis. A new subtype of Kounis syndrome?. Int J Cardiol.

[8] Dai B, Cavaye J, Judd M, Beuth J, Iswariah H, Gurunathan U (2022). Perioperative presentations of Kounis syndrome: a systematic literature review. J Cardiothorac Vasc Anesth.

[9] Kounis NG (2016). Kounis syndrome: an update on epidemiology, pathogenesis, diagnosis and therapeutic management. Clin Chem Lab Med.

[10] Desai R, Parekh T, Patel U (2019). Epidemiology of acute coronary syndrome co-existent with allergic/hypersensitivity/anaphylactic reactions (Kounis syndrome) in the United States: a nationwide inpatient analysis. Int J Cardiol.

[11] Tan PZ, Chew NW, Tay SH, Chang P (2021). The allergic myocardial infarction dilemma: is it the anaphylaxis or the epinephrine?. J Thromb Thrombolysis.

[12] (2015). Anaphylaxis and cardiovascular disease: therapeutic dilemmas. Clin Exp Allergy.

[13] Cheng TO, Kounis NG (2012). Takotsubo cardiomyopathy, mental stress and the Kounis syndrome. Int J Cardiol.

[14] Soufras GD, Kounis NG (2013). Adrenaline administration for anaphylaxis and the risk of takotsubo and Kounis syndrome. Int J Cardiol.

[15] Kounis NG, Mplani V, de Gregorio C, Koniari I (2023). Attack the ATAK; a challenging contemporary complex: pathophysiologic, therapeutic, and preventive considerations. Balkan Med J.

